# The Nanofication and Functionalization of Bacterial Cellulose and Its Applications

**DOI:** 10.3390/nano10030406

**Published:** 2020-02-25

**Authors:** Soon Mo Choi, Eun Joo Shin

**Affiliations:** 1 School of Chemical Engineering, Yeungnam University, Gyeongsan-si 38541, Korea; smchoi@ynu.ac.kr; 2 Department of Organic Materials and Polymer Engineering, Dong-A University, Busan 49315, Korea

**Keywords:** bacterial cellulose, microbial cellulose, sustainability, renewability, nanofication, functionalization

## Abstract

Since economic and environmental issues have become critical in the last several years, the amount of sustainable bio-based production has increased. In this article, microbial polysaccharides, including bacterial cellulose (BC), are analyzed as promising resources with the potential for applications in biofields and non-biofields. Many scientists have established various methods of BC production, nanofication, and functionalization. In particular, this review will address the essential advances in recent years focusing on nanofication methods and nanoficated BC applications as well as functionalization methods and functionalized BC applications.

## 1. Introduction

The 17 Sustainable Development Goals (SDGs) were formally established in September 2015 by the United Nations General Assembly and are intended to be achieved by the year 2030 with the goals classified into five subgroups: People, Planet, Prosperity, Peace, and Partnerships. Especially important are Sustainable Development Goals 12, “Ensure sustainable consumption and production patterns”, and 13, “Take urgent action to combat climate change and its impacts” [1]. The purpose of the SDGs is to develop solutions that enable and facilitate economic and societal development without environmental damage. This endeavor has focused on environmental protection by preventing the destruction of natural resources [2]. Recently, the bio-based production of chemicals has increased in prevalence [3,4,5]. Bacterial fermentative production from renewable resources may play an important role in SDGs 12 and 13, both environmentally and economically.

Various microorganisms play critical roles in the environment and green processes. Presently, a universal goal is the adoption of cleaner production and green technologies along with the preservation of natural resources [2]. Although microorganisms have plenty of advantages in sustainability, their representation remains poor in scientific research. This review article maintains that microorganisms, especially the materials derived from them are at the forefront of accomplishing the SDGs. 

Cellulose is one of the most abundant, renewable, and widely used natural polymers, which is commonly obtained from plant sources and has conventionally been employed for paper, textile, and pulp production, with a remarkable commercial reputation [6]. As previously stated, cellulose is well-known as a plant product, but some microbes, including algae, fungi, and diverse aerobic bacteria, are emerging as sustainable alternative sources of cellulose [6,7,8,9]. 

**Figure 1:**
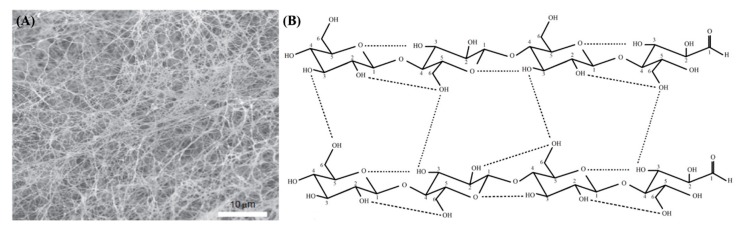
(**A**) SEM image of bacterial cellulose’s nanostructure and (**B**) Inter- and intra-hydrogen bonding of bacterial cellulose [22,23]. Copyright 2017 and 2014 Elsevier Ltd.

The shape of cellulose produced from bacteria (bacterial cellulose, BC) is the form of fiber with nano-sized width, similar to silk natural fiber produced from the silk worm (shown in Figure 1). In recent research, this BC tends to be reproduced as nano-sized particles with high crystallinity through acidic or enzymatic approaches. These processes are included into the “nanofication” of BC [10]. 

Bacterial cellulose (BC) was first reported as a white pellicle on a liquid medium during the acetic fermentations in 1886 [11,12]. This cellulose membrane was generated by *Bacterium xylimun*, which was renamed later to *Acetobacter xylimun* (*A. xylinum*) and known as *Komagataeibacter medellinensis*, with a 25 mm thickness [13,14]. Even if the BC is the same for plant cellulose in terms of molecules, it has drawn scientists’ attention due to its mechanical stability, thermostability, high crystallinity (70%–80%), high purity (free of lignin, hemicellulose, and pectin), low density, high specific surface area, excellent permeability, high porosity, high water content (up to 99%), and good biocompatibility [15,16,17]. Although BC is not entirely commercialized and is produced at a lab scale for research, its exceptional bioaffinity has stimulated the development of BC-based products, such as tissue-engineered scaffolds, wound-dressing materials, dental implants, artificial blood vessels, surgical mesh, bone fillings, heart valve, meniscus, artificial cartilage, etc. [18,19]. BC has also been employed in various food industries [20,21].

To accomplish the goal of emphasizing the importance, value, and understanding of BC, this employs four main sections. The following Section 1 the offers a basic introduction about BC; Section 2 describes the nanofication methods used to convert BC into both nanofibers and nanocrystals with specific characteristics, as well as their shape and applications. Section 3 focuses on the functionalization of BC, involving modification and hybridization, as well as its applications. The last section in this review article presents the conclusions with an overview. This article highlights the methods and applications of BC nanofication and functionalization, which have not been collected previously. 

### 1.1. Structure

Cellulose is an unbranched homopolysaccharide of a β-D-glucose linear chain linked with β-(1-4)-glycosidic bonds in a ^4^C_1_ conformation with the molecular formula (C_6_H_10_O_5_)*_n_*. Each glucose subunit of hydrogen bonds with neighboring glucose monomers within its chain (Figure 1). 

**Figure 2:**
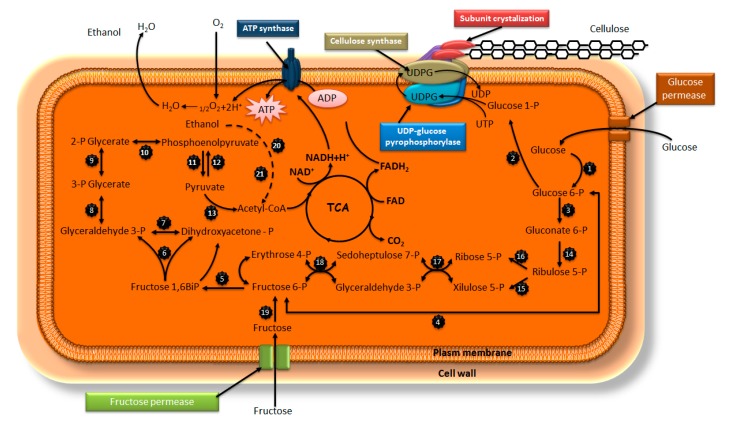
Hypothetical model of the pathway for the biosynthesis of cellulose by G. xylinus from exogenous sources: glucokinase-ATP (1); Phosphoglucomutase (2), glucose-6-phosphate dehydrogenase (3); Phosphoglucoisomerase (4); Fructokinase ATP (5), Aldolase (6); Triosephosphate isomerase (7); Glyceraldehyde 3-phosphate dehydrogenase (8); Phosphoglycerate mutase (9), enolase (10); Pyruvate kinase (11); Pyruvate biphosphate kinase (12), pyruvate dehydrogenase (13); 6-phosphogluconate dehydrogenase (14); Phosphorribulose epimeraase (15); Phosphorribulose isomerase (16); Transaketolase (17); Transaldolase (18); Fructokinase (19); Aldehyde dehydrogenase (20); Alcohol dehydrogenase (21) [28]. Copyright 1991 American Society for Microbiology.

The molecular structure of bacterial cellulose (BC) is the same as that of plant cellulose, except for its polymerization degree, which is 13,000 to 14,000 for plants and 2000 to 6000 for BC (for its macromolecular structure and properties) [24]. BC microfibrils were first established to be 100 times smaller than plant microfibrils in 1949 [25]. The microfibrous network is composed of well-arranged three-dimensional nanofibers, leading to the formation of a hydrogel membrane with both a high surface area and a high porosity. *Acetobacter xhylinum* creates cellulose I from a ribbonlike polymer and cellulose II from a thermodynamically stable polymer, as shown in Figure 2. During their biosynthesis, these bacteria uses saccharides as a carbon source (i.e., a nutritional medium), followed by polymerization into linear β-(1-4)-glucan chains before finally secreting their outer membrane through their cell wall. After that, the fibrils are assembled together forming nanofibril cellulose ribbons, resulting in the web network structure of BC, which gives it high porosity [26,27]. 

### 1.2. Biosynthesis and Its Mechanism

Gram-negative bacteria produce extracellular biomaterials. Among these biomaterials, only some are able to produce cellulose. Bacterial cellulose (BC) is produced by diverse species of bacteria belonging to Gluconacetobacter, as known as Acetobacter, Agrobacterium, Aerobacter, Achromobacter, Azotobacter, Rhizobium, Sarcina, Alcaligenes, Pseudomonas, Dickeya, Rhodobacter and Salmonella [27,29]. Acetobacter xylinum, which is known as the most efficient bacterial cellulose producer, is a Gram-negative and aerobic bacterium [26]. BC is produced by acetic acid bacteria in a nutritional culture medium via oxidative fermentation. Acetobacter xylinum absorbs diverse sugars, yielding cellulose in medium. It has an active metabolism at pH 3.0 to 7.0 and at temperature from 25 to 30 °C, using saccharides as a carbon source [25,30,31,32]. BC is produced during the air–liquid phase in a Hestrin and Schramm (HS) medium (a conventional culture medium), using glucose as a carbon source, peptone as a nitrogen source, yeast extract as a vitamin, and disodium phosphate and citric acid as a phosphate buffer for the medium [33]. The biosynthesis of BC by Acetobacter xylinum is the process that polymerizes glucose into linear β-(1-4)-glucan chains. The active site of BC synthase includes two uridine-diphosphoglucose precursor (UDP)-glucose binding sites and one β-(1-4)-glucan binding site [34], in which the hydroxyl at the C-5 of glucose is linked using UDP-glucose dephosphorylation and produces new links β-(1-4). The resulting β-glucan does not react with UDP-glucose, and the two new units of UDP-glucose are superimposed, inducing synthesis [35]. The cellulose chains allow new links with adjacent chains via the equatorially positioned OH intra and intermolecular hydrogen bonds, creating rigidity with a hydrophilic surface and a hydrophobic core together and producing non-soluble cellulose [36].

### 1.3. Cultivation Mode

Recently, the main culture mode employed for producing bacterial cellulose (BC) has been a static culture, which is a simple and widely used method that involves agitating/shaking the culture, thereby contributing to cost reduction, with the bioreactor culture providing suitable oxygen [9,37,38,39,40,41]. For either mode, the primary aim is to achieve high efficiency in BC production with an optimal form and appropriate properties for its application. The conditions of the culture mode are very important and impact the properties of the BC.

The static cultivation mode is comparatively simple with its low shear force and is an extensively applied method for producing BC pellicles. The medium is placed into shallow trays, inoculated, and cultivated for 1–14 days at a temperature from 28 to 30 °C and pH 4 to 7 until the cellulose almost fills the tray, since the production of BC relies on the area of the air/liquid interface [38,42]. BC made via this static method is of a hydrogel type with an excellent structure and properties. In this mode, BC hydrogel is produced in the air–liquid phase, where bound carbon dioxide is formed from the metabolism of the bacteria [42]. 

This mode is favorable if a predetermined form is required, such as for tissue engineering and regenerative medicine [37]. However, it may disturb its industrial/commercial application due to being time consuming and producing a low yield [43]. To overcome these hindrances, a fed-batch cultivation mode was proposed, which is a simple strategy used to enhance the yield to a proper level for commercialization and uses an alternative culture medium proposed by Shezad et al. [9]. 

Compared with the former static mode, an agitated/shaking cultivation mode facilitates cost reduction by increasing the yield of BC through continuously mixing oxygen into the medium. This process may create different shape and fibrous suspension of the spheres or pellets, according to the rotational speed [44]. It is difficult to form spheres with a rotational speed less than 100 rpm; irregular shapes are observed. Moreover, this method allows a low degree of polymerization, crystallinity, and problematic mechanical properties [45,46,47]. Numerous studies have demonstrated that an agitated/shaken culture may be the most appropriate mode for economic production despite the above-mentioned drawbacks [48].

Stirred-tank bioreactors are usually employed to produce fibrous BC suspensions that have low crystallinity, a low elastic modulus, and a low polymerization degree compared to the pellicle BC due to the shear stress of agitation [49], which controls the oxygen transfer and causes high energy consumption [50]. An airlift bioreactor, which is an alternative type of reactor, allows oxygen to transfer continuously within culture medium, leading to a proper oxygen supply [42]. This is more energy efficient and is accompanied by less shear stress compared to stirred-tank reactors [51]. 

## 2. The Nanofication of Bacterial Cellulose (BC) 

Bacterial cellulose (BC) has unique characteristics of high purity and high crystallinity and has thus been used as a basic source for the production of cellulose nanocrystals (CNCs) or cellulose nanowhiskers. Generally, it is accepted that the shape of nano-bacterial cellulose (NBC) is different depending on the source of its BC and the isolation methods used. The geometrical dimensions of CNCs are also important factors in the applications of NBCs. For example, rod-like CNCs offer significant advantages as drug carriers, as their cells internalize faster than those of spherical nanoparticles [52,53]. 

Well-known methods of BC nanofication are presented in Table 1, which are by acid, enzyme and ionic liquid. The general isolation process for bacterial cellulose nanocrystals (BCNCs) from BC is founded upon acid hydrolysis. The amorphous regions are favorable hydrolyzed, thereby improving the hydrolytic cleavage of their glyosidic bonds, which releases individual crystallites from the remaining intact crystalline regions with high acid resistance [54]. Commercially available enzymatic hydrolysis is also used for BCNCs to preserve the qualities of BC in its nanodimensional form [55].

### 2.1. Acid Hydrolysis

BC has been promising recently due to its environmentally friendly nature and higher purity [61], and it is a favorable starting material for producing CNCs with high crystallinity or cellulose nanowhiskers (CNWs) [56,59,60,64].

The main process for separating BCNCs from BC fibers is based on acid hydrolysis. The glucoside bonds in the amorphous or disordered portions of BC are first hydrolyzed by hydrogen ions, and the remaining crystalline regions with high acid resistance release individual crystallites [55].

**Figure 3:**
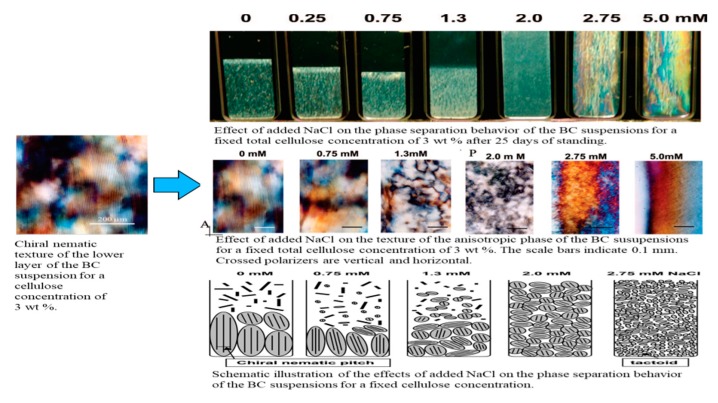
Schematic illustration of phase separation behavior of the bacterial cellulose (BC) suspensions. Reprinted with permission from Ref. [65]. Copyright 2009 American Chemical Society.

The phase separation of BCNC prepared by acid hydroylsis in water was investigated in detail by Asako Hirai et al. [65]. The authors reported that the suspensions separated into isotropic and chiral nematic phases under specific conditions of NaCl, which changed to an entirely liquid crystalline form; moreover, the overall chiral nematic domains were no longer observed at high concentrations of NaCl conductometric titration, as illustrated in Figure 3. 

Among inorganic acids, hydrochloric acid (HCl), sulfuric acid (H_2_SO_4_), and a mixture of HCl and H_2_SO_4_ are usually used. The properties of BCNCs from acid hydrolysis are shown in Table 2. 

The acidic conditions and BC sources strongly affect the efficiency of the yield, as well as the physical characteristics of nanocrystals, such as the hydrodynamic size, zeta potential, crystalline degree, and thermal stability behavior [66].

H_2_SO_4_ can rapidly hydrolyze due to its powerful hydrolytic action, although the hydrodynamic size of nanocrystal is relatively small (hydrodynamic size = 187 nm). However, H_2_SO_4_ produces highly negative-charged BCNCs by sulfonating the surface hydroxyl groups. The negative-charges of BCNC prevent its aggregation of nanocrystals driven by hydrogen bonding; then, the stable well-dispersed nanocrystal suspension can be obtained [69]. Marta Martinez-Sanz et al. [67] also studied whether the H_2_SO_4_ treatment of BC yielded CNWs. Their morphology consists of a cellulose I crystal allomorph and a nanofibrillar crystal, with a high aspect ratio in the range from 20 to 50, according to the applied hydrolysis conditions. The authors demonstrated that at least 48 h of H_2_SO_4_ hydrolysis time is needed to attack the amorphous regions and increase crystallinity, followed by neutralization to enable the production of highly crystalline BCNWs with a high aspect ratio and a thermal stability high enough to employ them as a reinforcement material in melt-compoundable composites. In contrast, HCl has milder hydrolytic action and generally generates cellulose crystallites with native crystalline structures that are free from charged groups, which results in low-charged and/or non-sulfated nanocrystals [68]. 

**Figure 4:**
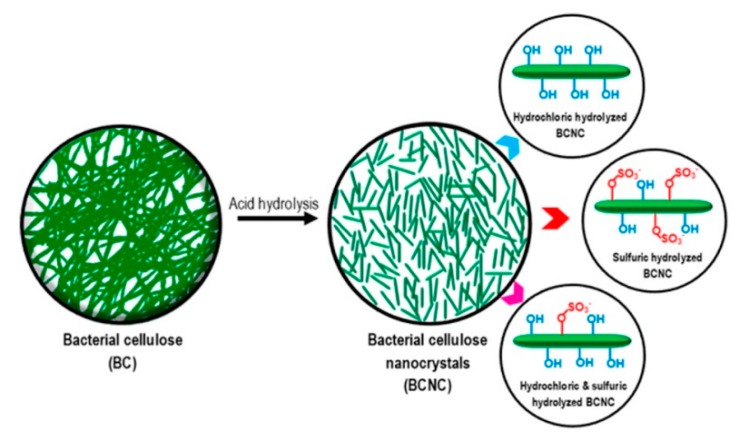
Diagram of bacterial cellulose nanocrystals (BCNCs) produced from BC by acid hydrolysis with diverse kinds of acid. Reprinted with permission from Ref. [66]. Copyright 2018 American Chemical Society.

These acid-hydrolyzed BCNCs can be helpful for applications requiring highly negatively charged to non-charged nanocrystals. This phenomenon is shown in Figure 4.

### 2.2. Enzymatic Hydrolysis

However, the acid hydrolysis method has been reported not only to reduce sulfate-containing nanocrystals with lower thermal stability than native cellulose [70] but also to significantly reduce the degree of polymerization (DP), thereby reducing the strengthening properties of the nanocomposites. Their thermal stability is a key parameter for their use as reinforcing fillers in the preparation of nanocomposites. [71]. Thus, new methods have been attempted to produce cellulose nanocrystals (CNCs) that retain the native structural properties of BC. Using a commercially available cellulase preparation is also a unique method. 

In recent years, the global trend of using lignocellulose biomass applications has been expanded to intermediate products, such as nanocellulose [72]. CNCs produced by enzymatic hydrolysis could be used to decrease their environmental impact. In recent years, several trade companies (e.g., Celluclast, Accelerase, Spezyme CP, and Viscoferm) have made efforts to increase the efficiency of cellulolytic enzymes using a process that is economically viable by improving the enzymes’ resistance to operational conditions, such as temperature and pH, or by increasing their speed of production and decreasing their price. 

The enzymatic hydrolysis mechanism involves amorphous domains with relatively large structural faults that act upon and transverse the cleavage of the microfibrils into short nanocrystals for the hydrolysis of cellulose. 

Endoglucanases randomly hydrolyze the amorphous parts of long cellulose chains to create smaller cellulose fragments [73], while single cellulose crystals are relatively less sensitive. [74]. In detail, these fragments include (i) endo-1,4-β-glucanases (EGs), which rapidly degrade the amorphous parts of the cellulose chains to create smaller cellulose fragments [73]; (ii) exoglucanases or cellobiohydrolases (CBHs), which generally attack the short crystalline regions of the cellulose and degrade cellulose by splitting off molecules from both ends of the chain, thus generating cellobiose dimers [75]; and (iii) β-glucosidases, which hydrolyze the cellobiose units that are produced during the CBH and EG attacks, converting them into glucose [76]. This phenomenon of the changeable susceptibility of the amorphous and crystalline parts of cellulose to enzymatic hydrolysis was used in this research to produce cellulose nanocrystals under buffer conditions.

Several studies have been conducted on the enzymatic hydrolysis of bacterial cellulose nanocrystals (BCNCs). Santa-Maria et al. [77] utilized cellobiohydrolase (Cel7A from Trichoderma reesei) in order to examine changes to the microstructure of the determined the effect of cellulose depolymerization and extended it to more complex cellulosic substrates and reaction conditions. Early in the reaction (approximately 30% hydrolysis), at high hydrolysis rates and high bound cellulase quantities, the untwisting of cellulose microfibrils was observed. As the hydrolysis reaction neared completion (>80% hydrolysis), largely thinned microfibrils (diameters of 3–5 nm) and channels (0.3–0.6 nm deep) along the lengths of the microfibrils were also observed.

George et al. [58] produced cellulose nanocrystals using cellulase (enzyme: *Trichoderma reesei* ATCC26921) under controlled conditions of time, temperature, and pH, as well as the properties of acid-processed nanocrystals. As a result, the nanocrystals fabricated by the compared enzymatic method were intimately related with better mechanical and thermal properties compared to the nanocrystals acquired from sulfuric acid hydrolysis. The enzymatically produced nanocrystals were parallel and more uniformly spaced in comparison to those produced with the acid process, which enhanced the crystals’ thermal and mechanical properties [72].

Domingues et al. [78] compared CNCs acquired from the acid hydrolysis of eucalyptus fibers to CNCs obtained upon the enzymatic hydrolysis of BC. They revealed the shape and surface chemistry of the two types of CNCs (acid and enzymatic hydrolysis CNCs: CNCa and CNCe). The shape of CNCe included axial grooves attacked by the enzyme along the axial direction and produced a C-shaped cross-section with zeta-potential values of −(23 ± 2) mV, which is different from the CNCa, which showed homogenous rod-shaped particles with zeta potential values of −(17 ± 1) mV at pH 6.

**Figure 5:**
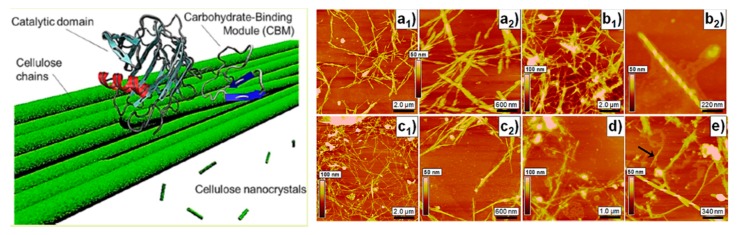
Process of enzymatic hydrolysis of bacterial cellulose left: mechanism of enzymatic hydrolysis, right: Atomic force microscopy height images of hydrolyzed BC after 74 h according to the following enzyme/BC ratios. Reprinted with permission from Ref. [79]. Copyright 2018 American Chemical Society.

Rovera et al. [79] described the kinetics of enzymatic hydrolysis experimental models with the correlated experimental conditions for BC to obtain nanocrystals, with turbidity in the yield of the nanocrystals, as shown in Figure 5. After this experiment, the authors recommended setting the hydrolysis process at a 2:1 enzyme/BC ratio up to 30 h of reaction or, alternatively, a 1:1 enzyme/BC ratio up to approximately 45 h. These temporal windows produce a yield approaching 25%, with a final morphology that mostly corresponds to the BCNCs.

Recently, Ricardo Brandes et al. [59] produced a BC using glycerol as a carbon source and isolated nanocrystals from BC using enzymatic hydrolysis. The authors also presented the most appropriate conditions for cellulose hydrogels, cellulase enzymes, and buffers to yield nanocrystals.

### 2.3. Shape of Hydrolyzed Bacterial Cellulose (BC) Nanocrystals

BC has been produced in many shapes and can be obtained with a highly hydrated (<99% water) network of highly crystalline, ribbon-like structures composed of microfibrils [80]. This material can have a very high surface area 20 times that of plant cellulose and outstanding mechanical properties [81,82,83].

Bacterial cellulose (BC) has a distinct ribbon-like 3D network structure (around 100 nm in diameter and around 100 μm in length). The geometrical dimensions of the produced bacterial cellulose nanocrystals (BCNCs) are about 10–50 nm in diameter and 100–1500 nm in length, depending on the sources of BC and the isolation methods. Longer lengths are classified as bacterial cellulose nanofibers (BCNFs) and shorter whiskers are classified as bacterial cellulose nanocrystals (BCNCs) (shown in Table 3).

**Figure 6:**
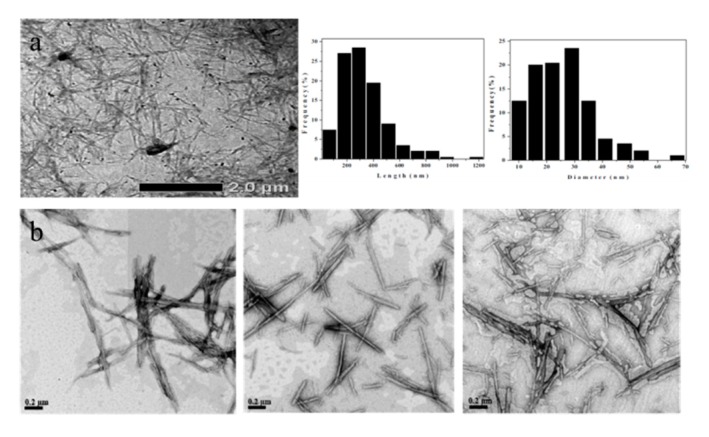
TEM image of rod-like CNC from bacterial cellulose, (**a**) particle size distribution. Reprinted with permission from Ref. [93]. (**b**)TEM micrographs of the BCNC prepared by different acid hydrolyses: HCl, H_2_SO_4_, HCl + H_2_SO_4,_. Reprinted with permission from Ref. [66]. Copyright 2015 Elsevier and 2018 American Chemical Society.

The shapes of the CNCs extracted from BC are different according to the conditions of hydrolysis. Rod-like nanofibers are the most common form resulting from acid hydrolysis, which includes the processes of centrifugation, dialysis, and sonication. Optimization was done by varying the variable for preparing the acid concentration, as well as the hydrolysis time, temperature, and acid BC ratio. Figure 6(a) shows the morphology of the CNCs with a rod-like structure whose length and diameter distribution has an average length of 325 nm and a diameter of 25 nm, with an average aspect ratio (L/D) around 13 [93]. Singhsa et al. [66] reported the morphology of bacterial cellulose nanocrystals (BCNCs) suspensions 100−700 nm in length and about 10−30 nm in as observed by TEM (shown in Figure 6(b)). The dimensions of the H_2_SO_4_-hydrolyzed BCNCs were more homogeneous than the HCl-hydrolyzed dimensions owing to their greater electrostatic repulsion of sulfate groups on the surface among the nanoparticles within the aqueous suspension.

Vasconcelos et al. [56] obtained BCNCs by acid hydrolysis from BC using different acid concentrations and reaction times and combined hydrolysis with H_2_SO_4_ and HCl, which were needle-like, with lengths ranging between 622 and 1322 nm, and diameters ranging between 33.7 and 44.3 nm.

Using an acid hydrolysis process, Pirich et al. [68] isolated needle-shaped BCNCs with diameters of 16–64 nm, fiber lengths of 258–806 nm, and a degree of crystallinity of 64%.

Recently, acid hydrolysis and mechanical treatment was used to produce nano-BC from BC. The results demonstrated that strong acids can remove amorphous parts and cut off cellulose chains into nanocellulose, whereas ultrasonic treatment produced smaller size particles and reduced particle agglomeration and homogenous dispersion products. This treatment produced nano-cellulose that was 99.99 nm in particle size, with 86.6% distribution. The produced nano-bacterial cellulose was β-cellulose [94].

Martínez-Sanz et al. [67] manufactured cellulose nanowhiskers extracted from BC and examined their various factors. The morphology of the bacterial cellulose nanowhiskers (BCNWs) was investigated by TEM, showing an expected decline in the nanowhiskers’ length with an increase in hydrolysis time. The aspect ratio of the BCNWs is a key parameter that conditions the reinforcing effect of the nanowhiskers when impregnated into a polymeric matrix. When this material is used as a higher reinforcement effecting agent, aspect ratios higher than 30 are common [95]. Nevertheless, these BCNWs revealed aspect ratios larger than 100, and the Young’s modulus reached a plateau corresponding to the maximum point of reinforcement [96].

### 2.4. Nanoficated Bacterial Cellulose (BC)-Based Nanocomposites

#### 2.4.1. Bacterial Cellulose (BC) Nanofibers-Based Nanocomposites

Bacterial cellulose (BC) nanofibers have several hydroxyl groups and are a hydrophilic material, displaying a surface area superior to that of plant cellulose due to being around 10–50 nm in diameter and 100–1000 nm in length. Many studies have shown through nanocomposites that bacterial cellulose can be used as a reinforcement for nanoscale dimensions and micrometer scale polymers as a substrate, which is well summarized in Table 4.

Juntaro et al. [97] developed a reinforced poly-l-lactic acid (PLA) matrix with sisal fibers-covered BC. First, the authors cultivated bacterial cellulose on sisal fibers as a substrate and determined a transparent pellicle covering the sisal fibers, which can improve the mechanical properties of BCNF/sisal fibers composites. Then, composited poly(l-lactic acid) (PLLA) with BC nanofibers covering the sisal fibers were shown to have an elastic modulus and tensile strength higher than the PLLA. Similar procedures to integrate BC-modified sisal fibers have been used for cellulose acetate butyrate matrices [106] and poly acrylated epoxidised soybean oil (polyAESO) [107] to improve the tensile moduli of nanocomposites.

BC nanofibers were employed as reinforcement for glass fiber epoxy composites [98]. Through the incorporation of 0.3 *wt* % BC nanofibers, the interlaminar fracture toughness for crack initiation and crack separation was improved by 128.8% and 111.0%, respectively.

To improve the mechanical properties of natural rubber, Phomrak et al. [99] reinforced the rubber using BC through the process of latex aqueous microdispersion. The hydrophilicity, opacity, and crystallinity of the composite were enhanced by incremental BC loading. The mechanical properties were effectively enhanced via the reinforcement by BC nanofibers, which had a Young’s modulus of 4128.4 MPa and a tensile strength of 75.1 MPa. Rubber/BC composites with good mechanical properties and thermal stability are likely to be used as rubber-based products or elastic packaging in many applications, including food and medical applications.

BC nanofiber-supported polyaniline (PANI) nanocomposites were synthesized by Wang et al. [100]. The resulting BC/PANI nanocomposites possessed an ordered flake-type nanostructure, which achieved a superb electrical conductivity of 5.1 S/cm and a high surface area of approximately 33.969 m^2^/g. The surface area measurements suggested that the BC/PANI composites accomplished an almost 11-fold enhancement compared to pristine BC fibers due to the fiber-like morphology being converted into an ordered flake-structure with the high densification and aggregation of BC/PANI flakes. By manipulating the ordered flake-type nanostructure, the BC/PANI nanocomposites achieved excellent electrical conductivity.

Yang et al. [101] described a novel facile and effective strategy to prepare micrometer-long hybrid nanofibers via the deposition of CdS nanoparticles onto the substrate of hydrated BC nanofibers with a well-defined hexagonal wurtzite structure, which is effective in photocatalysis. In addition, shape and size-controlled CdS cemented on renewable BC nanofibers was found to have potential as a recyclable photocatalyst in the area of catalytic processes.

#### 2.4.2. Bacterial Cellulose (BC) Nanocrystals-Based Nanocomposites

At the same nanocellulose concentration used for the reinforcing materials, cellulose nanofibers (CNFs) led to higher strength and modulus than did cellulose nanocrystals (CNCs) due to CNFs’ larger aspect ratio and fiber entanglement, but they had lower strain-at-failure because of their relatively large fiber agglomerates [108]. However, if the dispersibility of the nanocrystals is good, it is more preferred as a composite material.

George et al. [102] manufactured the nanocrystals after enzyme hydrolysis and entrapped the nanocrystals with a length in the range from 100 to 300 nm and a diameter in the range from 10 to 15 nm in polyvinylalcohol (PVA) polymer chains to generate polymer nanocomposites. This nanocomposite displays the excellent thermal stability of PVA due to high temperature bacterial cellulose nanocrystals (BCNCs) degradation, which starts at 379 °C. Additionally, the mechanical properties improved by adding a low concentration of BCNC and retained a tensile strength from 62.5 MPa to 128 MPa; the elastic modulus was noted to increase from 2 to 3.4 GPa.

Grunert et al. [103] prepared nanocrystals via the acid hydrolysis of bacterial cellulose composited with cellulose acetatebutyrate. The composites exhibited better thermal and mechanical reinforcement properties. Georgea et al. [105] manufactured hydroxypropyl methyl cellulose (HPMC)-based hybrid nanocomposite films with a unique combination of two nanomaterials, BCNCs and silver nanoparticles (AgNPs). The addition of BCNCs improved the hydrogen bonding interactions with HPMC and showed that AgNPs strongly interact with the hydroxyl groups of both HPMC and BCNCs, which makes it unavailable for other molecules, such as water, for interaction. The addition of BCNCs influenced the crystalline development of HPMC, whereas the AgNPs enhanced the overall crystallinity of the nanocomposite films. Seoane et al. [104] tested the disintegration of plasticized poly(hydroxybutyrate) (PHB) nanocomposite films containing bacterial cellulose nanocrystals reinforcement. The plasticized PHB nanocomposite film exhibited a rapid degradation, although nanocomposites with BC presented comparable disintegrable in composting to that of neat PHB due to the lower hydrophilic character of BCNCs.

### 2.5. Applications of Nanoficated Bacterial Cellulose (BC)-Based Nanocomposites

Emulsions are heterogeneous systems commonly composed of two immiscible liquids, one of which is dispersed as droplets in the other one. Conventionally, these liquid droplets, or the dispersed phase, are stabilized by small molecule surfactants or surface-active polymers, which can be adsorbed onto the interface, thereby decreasing the interfacial free energy. Emulsions can also be stabilized solely by fine solid particles to develop solid-stabilized or Pickering emulsions [109,110]. The reduction of hazardous surfactants and unavoidable environmental consequences let to the use of amphiphilic bacterial cellulose nanoparticles (BCNCs) as Pickering emulsions, which involved the irreversible adsorption of solid colloidal particles at the oil–water interface and stabilizing the emulsion droplets against coalescence by forming a mechanically robust monolayer [111,112].

**Figure 7:**
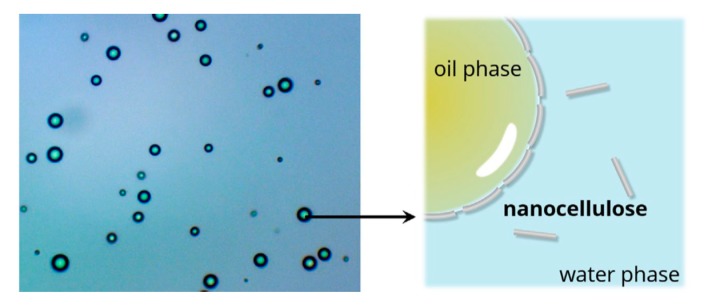
Scanning electron micrographs of Pickering emulsion stabilized by BCNCs. Reprinted with permission from Ref. [112]. Copyright 2017 Forestry and Forest Products Research Institute. Published by National Institute for Materials Science in partnership with Taylor & Francis.

Cellulose particles have been used to stabilize oil in water emulsions; however, their long lengths compared to the sizes of the droplets produced networks rather than individual drops, as shown in Figure 7. To improve this result, most studies have tried to reduce the size of the cellulose particles and have used hydrophobically modified cellulose particles.

Kalashnikova et al. [113] prepared cellulose nanoparticles via the hydrochloric acid hydrolysis of bacterial cellulose, whose characteristic BCNCs features include large aspect ratios and flat, ribbon-like cross-sections. These BCNCs present an elongated shape and a low surface charge density, allowing them to build a colloidal suspension in water. The BCNCs were determined to stabilize the hexadecane/water interface, initiating monodispersed oil in water droplets around 4 μm in diameter, which remained stable for some months.

The stability of Pickering emulsions is associated with particle concentrations and particle–particle interactions. The formation of a three-dimensional particle network at high particle concentrations can prevent the coalescence of dispersed droplets: the bridging of droplets by a monolayer of particles at a low particle concentration thus becomes the primary stabilization mechanism [114,115,116]. The weaker the cohesive force of the stabilized particles, the better the emulsion stability can be improved [117].

Yan et al. [118] manufactured oil-in-water Pickering emulsions with BCNCs, which exhibited a rod-like shape, high crystallinity, and good colloidal properties in an alginate solution for hydrophobic drug delivery. The characterization results revealed that BCNCs possessed good colloidal properties and could form a flocculated fibril network, which was able to help stabilize the Pickering emulsions. The irreversible adsorption of the BCNCs at the oil/water interface helped the Pickering emulsions preserve the droplets against coalescence. BCNCs are edible, non-toxic, biocompatible and biodegradable; accordingly, there are many studies in many fields on the emulsifiers of Pickering emulsions.

Yan et al. [119] compared BC and BCNCs as emulsifiers for Pickering emulsions. The BCNs with a crystallinity index (CrI) of 89.6%, an average size of 259.6 nm, a PDI (polydispersity index) of 0.26, and a zeta potential of about –34.8 mV exhibited better emulsifying performance than BC (microfibril shape and crystallinity index of 75.1%). Comparatively, BCNCs were more sensitive in terms of their pH response and ionic strength, exhibiting better colloidal properties. Accordingly, due to their lack of toxicity, high bioaffinity, and good biodegradability, emulsified BCNCs show promise for biomedical applications, such as in foods, cosmetics, and medicine.

The applications of BCNCs are mostly of interest to the paper and packaging industries to replace synthetic polymers with an environmentally friendly alternative. The main advantage of bacterial cellulose is predominantly its composition of pure cellulose, as no additional process is needed to extract lignin and hemicellulose. Furthermore, nanocellulose has an exceptional gas and water barrier at the nanoscale dimension. However, its highly hydrophilic character is responsible for its incompatibility with organic solvents and its poor adhesion to the hydrophobic surfaces of conventional polymeric materials. In order to improve this disadvantage, a hydrophobic polymer is adopted to improve the barrier to oxygen at a high relative humidity. Martinez-Sanz et al. [120] prepared BNCs and PLA composite film, which presented an excellent oxygen barrier at 70% relative humidity. BCNCs were also used as reinforcements for thermoplastic cornstarch films, which achieved relatively improved barrier and tensile properties, with a BCNC loading of 15 wt % [121]. These composite films using BCNCs as a reinforcing polymer have been shown to improve the gas barrier and mechanical properties for packaging purposes.

Given the attractive inherent properties of BCNCs, such as their high hydrophilicity, biocompatibility, and biodegradability, BCNCs are available for various biomedical applications involving tissue engineering, bioimiging, biosensing, and drug delivery. Singhsa et al. [66] demonstrated the potential of BCNCs in the biomedical field: in this study, the authors used BCNCs produced by acid hydrolysis as nucleic acid delivery systems. These BCNCs were completely complexed with siRNA at a weight ratio of 100, exhibiting their potential as vehicles in nucleic acid delivery. Pirich et al. [68] studied the influence of the sulfate esterification of BCNCs on their interactions with xyloglucan (XG). The results showed that self-assembled BCNCs-XG particles may be applied in various fields, including the enhancement of cellulose’s surface’s ability to immobilize biomolecules with a biosensor build up. Recent research may offer useful information related to producing rod-like BCNCs, which are bright biomaterials that can be generated from the BC and then modified to alter the characteristics of nanocrystals, depending on the desired area.

## 3. Functionalization of Bacterial Cellulose (BC)

In recent years, there has been an upsurge of interest in the practical use of bacterial cellulose (BC). This large spike related is by the increasing annual publications on BC [14]. The practical use of BC has become the focal point for studies on the functionalization of BC in particular, since functionalized BC allows the pioneering of materials with enhanced or novel properties via modifying or mixing multiple components. BC is a pure material (free of lignin and hemicellulose), as well as non-toxic, with high biocompatibility. However, it lacks proper functionalities to initiate cellular adherence and govern its porosity. In order to conquer these problems, researchers have tried to modify BC with chemicals, including modification and functionalization, as well as diverse in situ and ex situ methods. In this paper, we have distinguished between modification and functionalization, according to whether changes are done to the surface properties or to the bulk properties.

### 3.1. Suface Modification

The trends in biomedical application tend to prefer remarkable cellular responses, such as the adherence and proliferation of cells, which can then generate a neotissue. Related materials should exhibit good bioaffinity, a low immune reaction, and an improved tissue regenerating process. It has been reported that BC shows superb bioaffinity, mechanical properties, and water content. Bacterial cellulose (BC) assimilates nutrients in a culture medium and accordingly increases its cellular adherence and proliferation due to hydrophilicity, which involves absorbing nutrient media [120]. However, comparatively, BC shows cellular adherence and proliferation values lower than those of other protein-based materials [121]. This kind of drawback is surmountable for cellular attachment and proliferation via the modification of BC.

A number of modification strategies have been carried out to enhance the interactions with cells on various materials, including chemical grafting to a hydroxyl-rich surface and adding self-assembled monolayers (SAMs) [122,123,124,125,126,127,128]. Surface modification enables BC to offer novel applications, since this type of functionalization alters the properties of BC to improve its interactions in the body for biomedical use. In recent years, there has been an upsurge of interest in the practical uses of BC due to its high linear coefficient of thermal expansion (LCTE).

**Figure 8:**
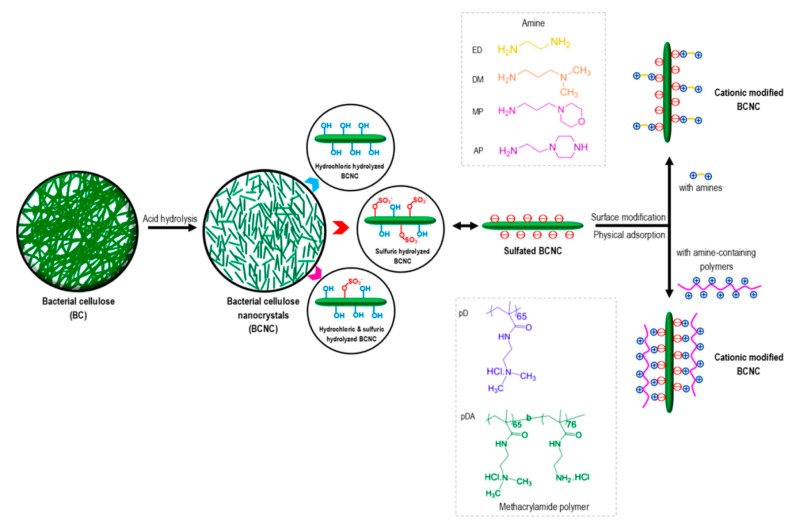
Schematic diagram of BCNCs Production from BC by acid hydrolysis with different types of acid and cationic surface modification of sulfated BCNC by physical adsorption techniques with amines and amine-containing polymers (methacrylamide polymers). Reprinted with permission from Ref. [66]. Copyright 2018 American Chemical Society.

There have also been frequent cellulose modification studies due to the compatibility between cellulose and hydrophobic thermoplastics, for hybridization, as cellulose’s hydrophilic nature can cause poor compatibility, which will then result in poor stress transfer efficiency between the matrix and the reinforcing fillers [129]. Therefore, a surface modification is necessary to alter the surface character of BC while exerting no effect on its bulk properties. In 2009, Lee at al. [130] reported that the hydrophilic surface of BC is converted into a hydrophobic surface by organic acid functionalization. The organic acid functionalized BC incorporated into PLLA caused a 50% enhancement of the tensile modulus, and a 15% enhancement of tensile strength in the nanocomposites. Also, the BC was observed to increase the thermal degradation temperature by 15 °C, with a higher storage modulus compared to pure PLLA. Accordingly, it was noted that the fabricated PLLA nanocomposites possessed enhanced properties via the surface functionalization of BC. Thus, it was established that BC that is modified chemically by grafting methyl terminated octadecyltrichlorosilane (OTS) or amine terminated 3-aminopropyltriethoxysilane (APTES) is able to increase the hydrophobic and electrostatic interactions with cells (Taokaew and Phisalaphong) [109]. In addition, BC has poor solubility due to the inter- and intra-hydrogen bonds present in its molecules. To overcome this, Rodriguez-Chanfrau et al. [131] conducted a chemical modification of BC via an acid treatment to enhance its potential application in regenerative medicine in 2017. For small peptides, this study showed several advantages compared to coating the surface of the polymer with proteins in terms of enzymatic degradation and immune inactivation. Rouabhia et al. used hybrid biomaterials immobilized with an arginine-glycine-aspartic acid (RGD) sequence to functionalize the polymer surface to enhance the interaction between the cells and biomaterials. The surface-modified BC membranes were obtained by immersing them in a solution of 0.4 M/L 3-aminopropyltriethoxysilane (APTES) in anhydrous toluene for 90 min. Then, the grafted cellulose membranes were soaked in dimethylformamide (DMF), followed by crosslinking. The prepared BC membrane functionalized by RGDC groups and modified by introducing gentamicin onto the surface was established to possess antibiotic properties [132]. Singhsa et al. [66] also developed cationized bacterial cellulose nanocrystals (BCNCs) via physical adsorption with amines and amine-containing polymers, as shown in Figure 8. Four different amines including ethylenediamine (EDA), N, N-dimethylethylenediamine (DM), 3-morpholinopropylamine (MP), and 1-(2-aminoethyl)-piperazine (AP) and two methacrylamide polymers with amine functional groups, were chosen to interact with the sulfated BCNCs.

Badshah et al. developed BC matrices modified by surface acetylation with a 1 mL mixture of acetic acid and sulfuric acid, followed by the addition of acetic anhydride. BC matrices with surface modification were explored for drug loading and release, and the study’s results demonstrated that this modification produced the desired properties [133]. Pertile et al. chose to modify the BC surface with plasma to enhance its cell affinity, for which lyophilized BC sheets were treated within a plasma reactor, and fed with N_2_ (100%), under the following conditions: time, 30 min; voltage, 425 V; current, 0.20 A; N_2_ flow, 10 sccm; pressure, 4 mbar. The MTS (3-(4,5-dimethylthiazol-2-yl)-5-(3-carboxymethoxyphenyl)-2-(4-sulfophenyl)-2H-tetrazolium) assay results showed that surface modification by nitrogen plasma enhances the adhesion of N1E-115 and Human Microvascular Endothelial Cells (HMEC-1) cells by a factor of two for HMEC [134].

### 3.2. Fuctionalization and Hybridization

Although bacterial cellulose (BC) possesses unique characteristics [14,15,16], several properties hinder its application in the biomedical area. For example, it is difficult to absorb in the body and has a dense form in its dried state [135]: it also has no antibacterial characteristic [136]. Oliveira Barud et al. [137] fabricated BC-based sponge-like nanocomposites by soaking BC membranes in silk fibroin solutions at concentrations of 1%, 3%, and 7%. The prepared BC/SF nanocomposites improved the material’s biocompatibility and ability to induce cell adhesion, resulting in a non-genotoxic material that was safe for medical applications, by introducing a silk fibroin with amino acids acting as cell receptors and mediating interactions with cells. Wang at al. [138] proved that a BC hydrogel hybridized with gelatin can support cell growth and proliferation, yielding excellent biological compatibility; the authors concluded that this hydrogel would be a good candidate for use with tumor cells cultured in vitro for cancer biology studies, clinical diagnosis, and tumor tissue engineering applications. BC functionalized by kaolin particles was developed to improve biomaterial performance, especially to control the rate at which blood clots for collagen regeneration, since kaolin can influence the rate of blood clotting; clotting is promoted by the surface charges of kaolin particles, which possess antibacterial properties. Lin et al. successfully developed BC–chitosan membranes with a molecular weight of 30 kDa and degree of deacetylation of 90% by immersing wet BC membranes in 0.6% chitosan solution for 12 h, followed by freeze-drying. The results for the water swelling, contents, retention, and permeability experiments showed that the BC–chitosan membranes possessed balanced functionality according to the water uptake and dehydration, helping them maintain proper moisture content for wound healing [139]. In addition, silver-functionalized BC was developed with silver nanoparticles deposited on nanofibrillated BC by the photochemical reduction process using UV radiation. An Ag/BC hybrid composite exhibited antibacterial activity and stability in a moist environment, which may assist wound healing [140]. In addition, ZnO particle-incorporated BC sheets were prepared by ultrasonic-assisted in situ synthesis, which is advantageous for forming nanocrystalline ZnO particles without destroying the 3D structure of the BC. It was determined that the sizes of the ZnO particles were close to the diameters of the BC nanofibrils, and the BC-based nanocomposites showed excellent antibacterial activity against both Gram-positive and Gram-negative bacteria [141]. Zhijiang et al. prepared a soy protein nanoparticle-modified BC electrospun nanfiber scaffold via the ultrasound-induced self-assembly technique. To fabricate the modified BC scaffold, BC nanofibrous scaffolds were immersed in a soy protein solution and ultrasonicated for the ultrasound-induced self-assembly process. The modified BC nanofibers had a diameter from 80 to 360 nm with soy protein nanoparticles self-assembled on the surface. The resulting data show greater biocompatible than pure BC nanofibers, owing to the soy protein nanoparticles layer and resulting in improved cellular interactions [142].

### 3.3. Applications of Fuctionalized BC

Certain modifications of BC are unavoidable for various applications. Several changes allow one to improve BC in terms of its physicomechanical and surface characteristics. Diverse types of modification have been reported using polymers, proteins, solvents, and biosynthetic strategies in order to fabricate advanced BC-based materials with specific properties. For applications related to drug delivery, BC matrices with surface acetylation combined via freeze-drying have been used to balance the properties associated with controlled drug release. Badshah et al. concluded that surface-modified BC has the potential for use in drug delivery systems, particularly in prolonged and controlled drug delivery [133]. In addition, Singhsa et al. evaluated cationized bacterial cellulose nanocrystals (BCNCs) as nucleic acid delivery systems by not only examining the cationic-modified BCNC-siRNA complexation, but also the cytotoxicity in HeLa cells, derived from cervical cancer cells, which were prepared through simple cationic surface modification via physical adsorption with the chemicals and polymers enclosing functional amine groups. This is a promising biomaterial that has been modified to alter the properties of BCNCs depending on the required area [66]. Soy protein nanoparticles surface-modified BC electrospun nanofiber membrane scaffolds were developed by the ultrasound-induced self-assembly technique, which is more bioactive and promising than bone tissue engineered scaffolds [75]. Gentamycin is also a widely used drug in the treatment of bone infection, and it is a substance designed for use in bone recovery. Dydak et al. [143] reported that gentamycin-modified BC showed very low cytotoxicity and was able to inhibit the proliferation of the bone pathogen, S. aureus. This modification strategy allows us to meet the demands of implantology and has high suitability for orthopedic applications. In addition, BC’s surface characteristics can be changed via plasma treatment approaches. The effects obtained are available by controlling several parameters, including the gas and reaction conditions. In this work, the treatment of nitrogen plasma allowed us to enhance the concentration of functional groups on BC surface in a stable way along with time, accordingly improving the adherence of cells to the BC materials and revealing the material’s potential for used in tissue engineering applications [10].

## 4. Conclusions and Overview

BC is attractive for use in various applications due to its mechanical stability, thermostability, high crystallinity (70%–80%), high purity (due to being free of lignin, hemicellulose, and pectin), low density, high specific surface area, excellent permeability, high porosity, high water content, and biocompatibility. Especially, compared with the nanocellulose produced from lignocellulose, nanocellulose produced from bacterial cellulose (BC) is advantageous for hybridization due to its high purity, low density, high specific surface area, and high crystallinity, resulting in the excellent mechanical and thermal properties of the nanocomposites. However, its drawbacks include struggling to delay degradation, high hydrophilicity (resulting in cellular adherence and proliferation lower than other protein-based materials), and poor compatibility with other hydrophobic polymers as it is used as a reinforcing filler. This article highlighted relevant studies to determine successful strategies for the nanofication and functionalization of BC to enhance its functionality and maximize the balance of its advantages and disadvantages and highlight its promising uses in various applications. Although BC has been still studied to examine the industrial availability, its use in numerous applications including modern food, the health industry, bioengineered organ/tissue, drug delivery, and renewable materials, have been expected in the next few years, along with the current and future progresses of science and technology.

## Figures and Tables

**Table 1:** Nanofication procedure of bacterial cellulose.

Main Process	Purification	Treatment Procedure	Post-treatment	References
Acid hydrolysis	Washing, homogenization, drying, grinding	H_2_SO_4_/HCl mixture at 45 °C, dilution	Centrifugation, dialysis, ultrasonication	Vasconcelos et al. [56] Revol et al.[57] George et al.[58]
Enzymatic hydrolysis	Mechanically defibrillating	Cellulase and citrate buffer solution at 50 °C under shaking until colloidal suspension was observed	Centrifugation, dialysis	Brandes et al.[59] Moriana et al.[60]
Ionic liquid	Freeze-dried for 48 h	1-ethyl-3-methylimidazolium acetate (EMIMAc) ILs at 90 °C under vacuum to remove water traces	Centrifugation	Raghuwanshet al. [61] Young et al. [62] Bowron et al.[63]

**Table 2:** The properties of BCNCs from acid hydrolysis.

Acid Type	Raw Bacteria	Yield (%)	Crystallite Size (nm) *	Crystallinity Index (%) **	Zeta Potential (mV)	References
H_2_SO_4_	Komagataeibacter xylinus	78.6~81.5	6.3	85~87	−(31.5 ± 1)	[66]
	Gluconacetobacter xylinum 7351	-	1.04~1.74	77~90	-	[67]
	Nata de coco by Komagataeibacter xylinus	14~63	5.11~5.93	79~92	−(24.7 ± 2.1~ 53.6 ± 0.7)	[56]
	Acetobacter xylinum	-	5.5~7.33	82	−(46 ± 1)	[68]
HCl	Acetobacter xylinum	-	5.41~7.41	83	−(5 ± 1)	[68]
	Komagataeibacter xylinus	84.4~85.6	6.5~6.7	87~89	−11	[66]
	Nata de coco by Komagataeibacter xylinus	14	5.22	83	−(43.9 ± 0.8)	[56]
HCl + H_2_SO_4_	Komagataeibacter xylinus	80.5~82.4	6.4~6.6	87~89	−(25 ± 1)	[66]
	Acetobacter xylinum	-	5.32~7.94	82	−(40 ± 1)	[68]

* The CS was determined using the Scherrer equation as following: CS = $\frac{K\lambda }{\beta cos\theta }$, where K is the shape factor (0.9), λ is the X-ray wavelength (1.54 Å), β is the full width at half-maximum (fwhm), and θ is the Bragg angle. ** The crystallinity index (CI) and crystallite size (CS) were calculated based on XRD measurements. CI was calculated from the following equation: CI (%) = $\frac{I_{200}-I_{am}}{I_{200}}$ × 100, where I200 is the overall intensity of the peak at 2θ = 22.7° and Iam is the intensity of the baseline at 2θ = 18°.

**Table 3:** Geometrical domain scale and properties of cellulose nanocrystals (CNCs) according to sources.

Cellulose Source	Degree of Polymerization (DPw)	Length (nm) [4,5]	Cross-section (nm)	Crystallinity (%)
Algal [84]	3–5	>1000	10–20	95
Bacterial [85,86,87,88,89]	2000–6000	100–1500	5–10 by 30–50	84–89
Cotton [87,88,89,90]	8000–15000	200–350	5	40–60
Tunicate [91,92]	900–3500	100–1000	10–20	50–60

**Table 4:** Improved mechanical properties of nanocomposites reinforced with bacterial cellulose (BC) nanofibers and nanocrystals.

Reinforcement	Substrate	Improved factor			Reference
		Factors	Before Improvement	After Improvement	
BC nanofiber covered sisal fibers	poly(L-lactic acid) (PLLA)	Tensile modulus (MPa)	64	113.8 ± 8.10	Juntaro et al. [97]
		Young’s modulus (GPa)	2.5	11.21 ± 0.69	
BC nanofiber	Glass fiber	Crack initiation (%)	100	128.8	Vu et al. [98]
		Crack separation (%)	100	111.0	
	Natural rubber	Tensile modulus (MPa)	0.8 ± 0.1	75.1 ± 27.1	Phomrak et al. [99]
		Young’s modulus (GPa)	0.0016 ± 0.4	4.13 ± 0.99	
	Polyaniline (PANI)	Electrical conductivity (S/cm)	1.61 × 10^−4^	5.1	Wang et al. [100]
	CdS particles	Reaction rate of photocatalyst (min^−1^)	0.00013	0.012	Yang et al. [101]
BC nanocrystals	Polyvinylalcohol (PVA)	Tensile strength (MPa)	62.5	128	George et al. [102]
		Elastic modulus (GPa)	2	3.4	
		Melting temperature (°C)	203.3	212.7	
	Cellulose acetate butyrate	Melting temperature (°C)	146.5	149.3	Grunert et al. [103]
		Elastic modulus (GPa) at 81°C	0.9	1.5	
	Poly(hydroxybutyrate) (PHB)	Contact angle (^o^)	76	72	Seoane et al. [104]
		Disintegration at 14 days (%)	18	50	
BC nanocrystals+ silver nanoparticles (AgNPs)	Hydroxypropyl methyl cellulose	Tensile strength (MPa)	59 ± 5.3	78 ± 6.9	Georgea et al. [105]
		Tensile modulus (GPa)	1.33 ± 0.25	2.28 ± 0.27	
		Moisture sorption (%), Iglesias and Chirife model	3.37 ± 0.91	2.18 ± 0.91	
